# Knowledge Retention of the NIH Stroke Scale among Stroke Unit Health Care Workers Using Video vs. E-Learning: Protocol for a Web-Based, Randomized Controlled Trial

**DOI:** 10.3390/healthcare9111460

**Published:** 2021-10-28

**Authors:** Avinash Koka, Mélanie Suppan, Emmanuel Carrera, Paula Fraga-Freijeiro, Kiril Massuk, Marie-Eve Imbeault, Nathalie Missilier Perruzzo, Sophia Achab, Alexander Salerno, Davide Strambo, Patrik Michel, Loric Stuby, Laurent Suppan

**Affiliations:** 1 Division of Emergency Medicine, Department of Anesthesiology, Clinical Pharmacology, Intensive Care and Emergency Medicine, University of Geneva Hospitals and Faculty of Medicine, 1211 Geneva, Switzerland; 2 Division of Anesthesiology, Department of Anesthesiology, Clinical Pharmacology, Intensive Care and Emergency Medicine, University of Geneva Hospitals and Faculty of Medicine, 1211 Geneva, Switzerland; melanie.suppan@hcuge.ch; 3 Stroke Center, Department of Neurology, Geneva University Hospitals and Faculty of Medicine University of Geneva, 1211 Geneva, Switzerland; emmanuel.carrera@hcuge.ch (E.C.); Nathalie.Peruzzo-Missillier@hcuge.ch (N.M.P.); 4 Stroke Center, Neurology Service, Department of Clinical Neurosciences, Lausanne University Hospital, 1011 Lausanne, Switzerland; Paula.Fraga-Freijeiro@chuv.ch (P.F.-F.); Kiril.Massuk@chuv.ch (K.M.); Marie-Eve.Imbeault@chuv.ch (M.-E.I.); Alexander.Salerno@chuv.ch (A.S.); Davide.Strambo@chuv.ch (D.S.); patrik.michel@chuv.ch (P.M.); 5 Specialized Facility in Behavioral Addictions ReConnecte HUG, 1211 Geneva, Switzerland; sophia.achab@hcuge.ch; 6 WHO Collaborating Center in Training and Research in Mental Health, UniGe, 1211 Geneva, Switzerland; 7 Genève TEAM Ambulances, Emergency Medical Services, 1201 Geneva, Switzerland; l.stuby@gt-ambulances.ch

**Keywords:** NIHSS, e-learning, video, NIHSS certification, stroke, stroke unit, medical education, continuous education

## Abstract

The National Institutes of Health Stroke Scale (NIHSS) is commonly used to triage and monitor the evolution of stroke victims. Data regarding NIHSS knowledge in nurses and physicians working with stroke patients are scarce, and a progressive decline in specific knowledge regarding this challenging scale is to be expected even among NIHSS certified personnel. This protocol was designed according to the CONSORT-eHealth (Consolidated Standards of Reporting Trials) guidelines. It describes the design of a randomized controlled trial whose primary objective is to determine if nurses and physicians who work in stroke units improve their NIHSS knowledge more significantly after following a highly interactive e-learning module than after following the traditional didactic video. Univariate and multivariable linear regression will be used to analyze the primary outcome, which will be the difference between the score on a 50-question quiz answered before and immediately after following the allocated learning material. Secondary outcomes will include knowledge retention at one month, assessed using the same 50-question quiz, user satisfaction, user course duration perception, and probability of recommending the allocated learning method. The study is scheduled to begin during the first semester of 2022.

## 1. Introduction

### 1.1. Background

Stroke is a frequent and time-critical emergency associated with significant morbidity and mortality [1,2]. Even though relative stroke incidence and mortality have declined since 1990, the expansion of the global population has resulted in an overall increase in the absolute number of strokes [3]. In the context of the COVID-19 pandemic, an association between SARS-CoV-2 infection and stroke has been described, with infected patients being at higher risk of worse functional outcomes and even death [4]. Efficiently assessing stroke victims is therefore more important than ever to limit the high morbidity burden associated with this pathology. Indeed, some interventions, such as intravenous thrombolysis and thrombectomy, improve functional and survival prognosis after stroke [1]. As these treatments are more effective when performed rapidly, cerebral imagery must be promptly obtained after patient admission. Scanners and Magnetic Resonance Imaging units are, however, often overloaded and overbooked [5,6], and delays in obtaining cerebral imagery are associated with worse functional and survival outcomes [7]. Adequate and timely triage is therefore mandatory, and most guidelines recommend using the National Institutes of Health Stroke Scale (NIHSS) to triage stroke victims for revascularization treatments [5,8]. Both patients with very mild [9] and very severe stroke [10] have their particularities of presentations, causes, and outcomes. Moreover, the NIHSS is also used in many stroke units to monitor the patients’ evolution [11,12], with neurological monitoring every 6 h being mandatory in Swiss stroke units in the initial surveillance phase after stroke [13]. It is therefore critical that stroke unit personnel master the application of this scale, as interpersonal variability in its interpretation can lead to inappropriate diagnostic or therapeutic procedures [14].

Digital learning resources play an important role in education today [15]. Over the last decade, e-learning modules have become popular in health professional education [16,17]. Medical and paramedical personnel often work in shift patterns, thus greatly limiting their availability for traditional teaching sessions. E-learning modules are very helpful in this setting, as the content can be viewed without time constraints, can be interrupted and resumed as needed, and can be highly interactive. Self-paced highly interactive e-learning modules have been shown to improve user satisfaction [18,19].

Traditionally, the NIHSS has been taught using a didactic video created by Dr. P. Lyden [20], which is freely available on the internet [21]. Although usually effective in teaching medical procedures [22], videos lack interactivity, making them less engaging than interactive learning materials. However, engagement has been shown to improve knowledge acquisition [23,24].

To enhance learner engagement, we have created a highly interactive electronic learning (e-learning) module using Storyline (Articulate Global, Inc., New York, NY, USA). This module was shown to be more effective than the traditional didactic video in paramedics [18] and in medical students [19]. However, the two prior studies assessing the impact of this e-learning module were carried out in populations naïve to the use of this scale. Moreover, these studies were not designed to determine an effect on knowledge retention.

With time, nurses and physicians working in a stroke unit might have forgotten or overlooked certain key aspects of the application of the NIHSS. We hypothesize that our e-learning module might be more effective in reminding them of the principles underlying the application of the NIHSS than the traditional didactic video. We furthermore hypothesize that knowledge retention at one month should be higher after following this module.

### 1.2. Objectives

Our primary objective is to determine if healthcare workers belonging to a stroke unit improve their NIHSS knowledge more significantly after following a highly interactive e-learning module than after following the traditional didactic video.

The secondary objectives are to perform a cross-sectional description of actual NIHSS knowledge in these specific wards and to determine whether following either training material allows better retention of knowledge at one month.

## 2. Materials and Methods

### 2.1. Study Design and Setting

**Figure 1:**
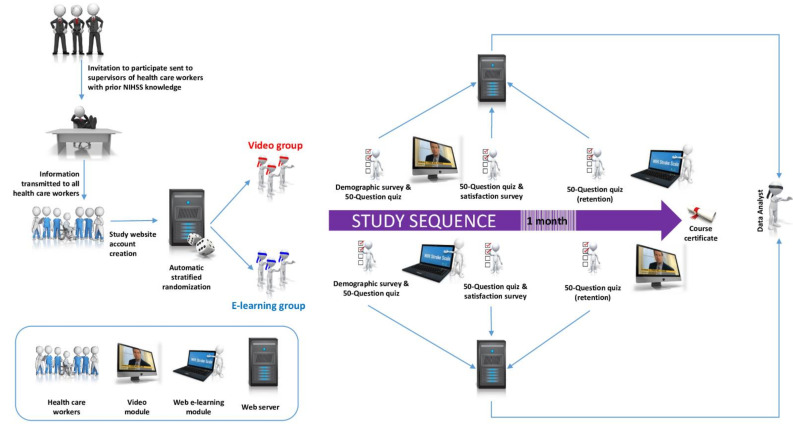
Study design.

A prospective, multi-centric, web-based, triple-blind (participants, investigators, data analyst) randomized controlled trial will be carried out following the CONSORT-eHealth guidelines [25] and integrating elements from the Checklist for Reporting Results of Internet E-Surveys (CHERRIES) (Figure 1) [26].

### 2.2. Online Platform

An internet-based and GDPR (General Data Protection Regulation)-compliant study platform will be created using the Joomla 3.9 content management system (Open Source Matters, Inc., New York, NY, USA) [27]. Randomization will be stratified according to professional status (nurse or resident physician), prior NIHSS knowledge (limited, moderate, or extended), and institution by virtue of specific links displayed on the front page. Clicking on the appropriate link will automatically randomize the participant into one of two groups (video or e-learning) through the use of Gegabyte’s Random Article module [28]. A specific and straightforward registration form (Membership Pro, Joomdonation [29]) will then be displayed. Participants will have to enter and confirm a valid e-mail address, choose a secure password, and validate a captcha field. After clicking on the “Register” button, a generic email containing a data policy statement will be sent to the email address entered by the participant. Simultaneously, the participant will be automatically logged in and a first questionnaire will be displayed.

### 2.3. First Questionnaire

This questionnaire (Table 1) is designed to gather all relevant demographic information regarding the participant and to obtain data regarding their prior NIHSS training and knowledge. It will be administered using the Community Surveys 5.6 component (CoreJoomla) [30].

Regular expression (Regex) rules will be used to avoid invalid data entry. A 5-point Likert scale (very low/not at all to very high/very much) will be used to record appropriate data.

After completing this first questionnaire, participants will be redirected to a first 50-question quiz designed to assess their baseline knowledge. This quiz is identical to the one used in our previous studies [18,19]. After completing this quiz, participants will be shown the training material they have been allocated to.

### 2.4. Learning Material

**Figure 2:**
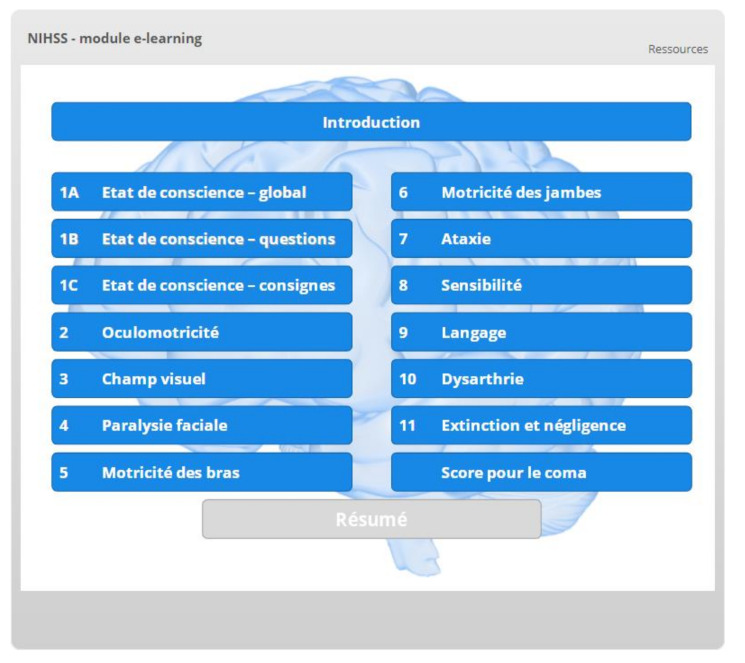
Table of contents of the interactive e-learning module.

The control group will follow the traditional didactic video created by Dr. P. Lyden [20], USA, which we have subtitled in French [31]. Meanwhile, the e-learning group will be presented with version 21c of our e-learning module [32]. This module was created using Storyline 3 (Articulate Global Inc., New York, NY, USA) and contains 184 interactive slides. Its structure follows the logic supporting the NIHSS (Figure 2).

**Figure 3:**
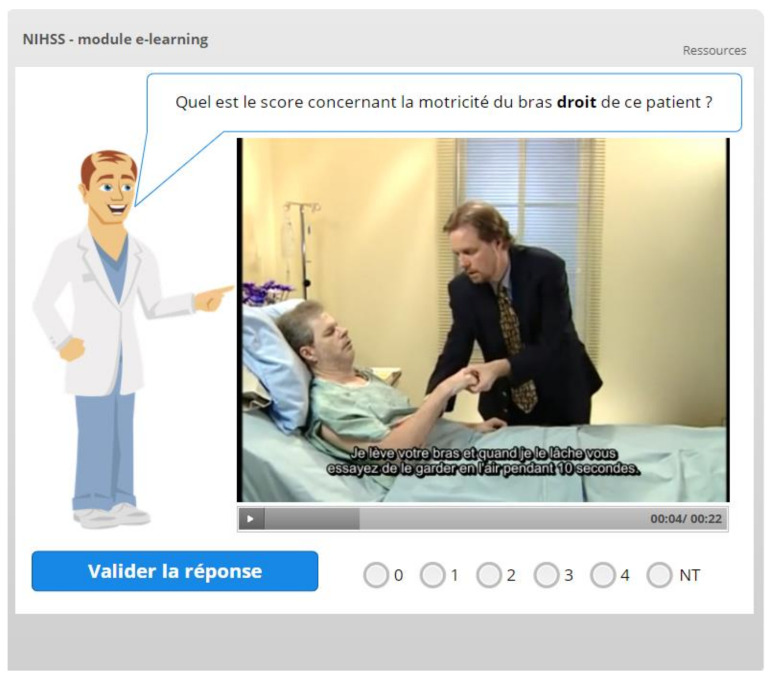
Embedded video and quiz interaction.

Participants are required to go through all 12 chapters before reaching the final chapter, which summarizes the entire NIHSS score. Video extracts taken from the original didactic video have been embedded in each chapter (Figure 3), as their presence has been shown to improve knowledge acquisition [19].

**Figure 4:**
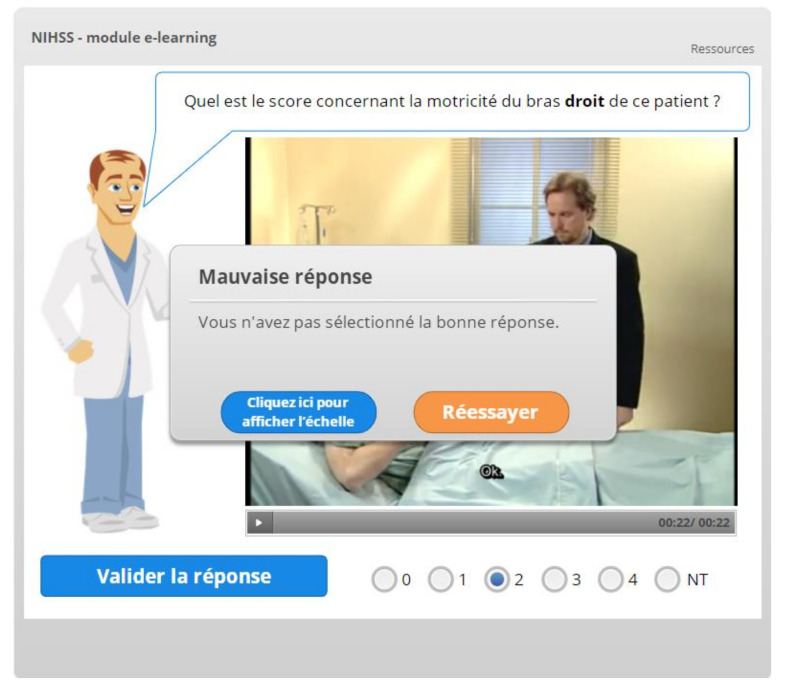
Feedback displayed after a wrong answer.

In this module, quizzes and feedback [33,34] are used extensively to enhance learner engagement and promote knowledge acquisition (Figure 4). Whenever the learner gives a wrong answer, the feedback message includes the possibility to review the scoring logic related to the specific NIHSS item being tested.

**Figure 5:**
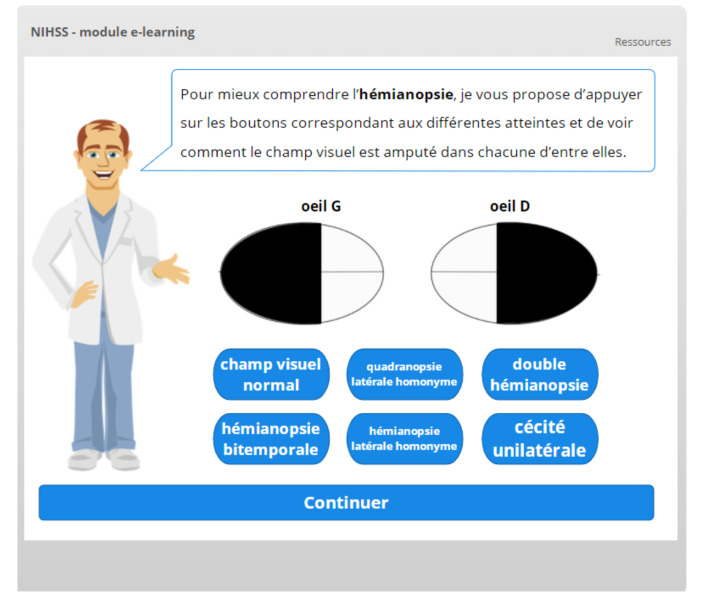
Interactive slide used to remind or teach visual fields.

**Figure 6:**
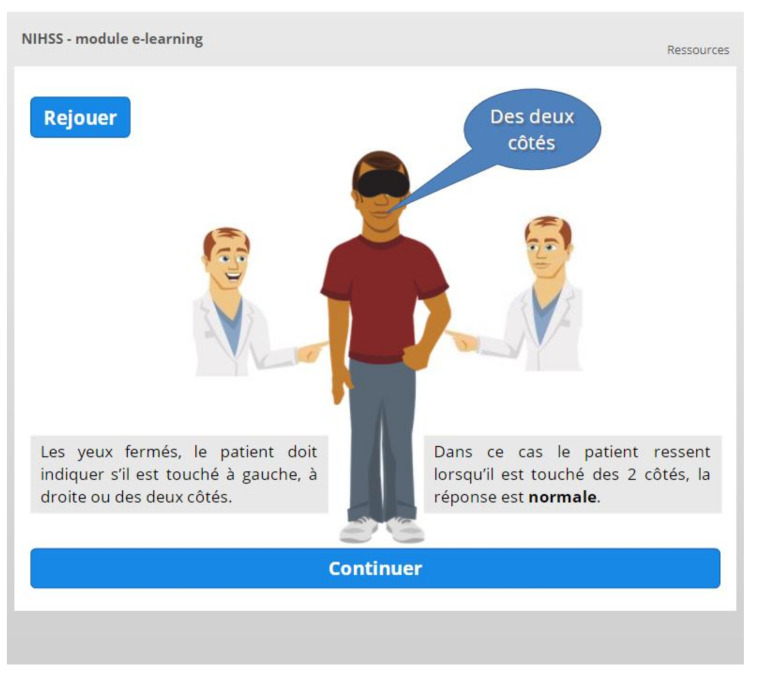
Interactive slide used to remind or teach the concept of extinction.

Depending on the complexity of the item, specific animations or interactions are used to facilitate knowledge acquisition. This is for example the case for visual fields (Figure 5) and for extinction/inattention (Figure 6).

After completing the learning material, participants will be asked to answer the same 50-question quiz to determine their knowledge acquisition. At the end of this quiz, a post-course satisfaction questionnaire will be displayed (Table 2).

### 2.5. Knowledge Retention

One month (28 days) after completing the second 50-question quiz, participants will receive an email inviting them to test their knowledge retention. After completing this last questionnaire, they will be awarded a course completion certificate. There will be no other incentive to promote their participation.

### 2.6. Outcomes

The primary outcome will be the difference between the score on the 50-question quiz answered before and after following the allocated learning material. Secondary outcomes will be the overall performance in the pre-course 50-question quiz according to profession and prior NIHSS experience, performance in the same quiz undertaken one month after completion of the learning material (knowledge retention), time to course and quiz completion, user satisfaction with the learning method, user perception of the duration of the course, and probability that the user would recommend the learning material they have been allocated to a colleague.

### 2.7. Participants and Sample Size

Seventy-two participants are required to have 80% chance of detecting a difference of 2 points in the post-course 50-question quiz between groups at the 5% significance level. A total of 120 invitations should be sent, and a participation rate of 60% will therefore be required.

Disclaimers and data policy statements will be displayed on the front page and on the registration page (through a link that will open these statements in a modal).

### 2.8. Data Curation and Statistical Analysis

Stata 16 (StataCorp LLC, College Station, TX, USA) will be used for data curation and statistical analysis. Data will be curated by one author L.S. (Laurent Suppan), who will assign neutral names randomly to the e-learning group and the video group. The curated DTA file will then be transferred to another author L.S. (Loric Stuby) for data analysis.

A univariate linear regression will be used to analyze the primary outcome. Then, a multivariable linear regression model will be generated with profession, prior NIHSS knowledge, and work center as adjustment variables. The conditions of application will be checked, i.e., the residues distributions normality assessed graphically on a histogram and the homoscedasticity using the residual-versus-fitted plot function in Stata.

The analysis of the same quiz undertaken one month later (knowledge retention) will follow the same procedure as the primary outcome.

The assessment of the distribution of the other continuous variables, i.e., time for course completion and time for quiz completion, will be done graphically and using the Shapiro–Wilk test in case of doubt. Data will be described as means (95%CI) or median (Q1–Q3) depending on what is applied. Then, a Student’s unpaired t-test or the Mann–Whitney U test will be applied.

The cross-sectional assessment of the performance in the pre-course 50-question quiz will be described overall and according to profession and prior NIHSS experience.

A sensitivity analysis will be performed by excluding those who have previously followed either the e-learning module or the video from Patrick Lyden [20].

User satisfaction, user course duration perception, and probability of recommendation will be assessed using a 5-point Likert scale and analyzed graphically, then using the Fisher’s exact test.

The data file will be uploaded to the Mendeley Data repository.

### 2.9. Ethical Considerations

The study protocol was submitted to the regional ethics committees of both university hospitals (Req-2021-00543), which waived the need for ethical approval as this study falls outside of the scope of the Swiss Human Research Act from 2011.

The study is scheduled to begin during the first semester of 2022.

## 3. Discussion

### 3.1. Main Considerations

This prospective, multi-centric, web-based, triple-blind (participants, investigators, data analyst) randomized controlled study comparing video-based and e-learning should help determine if a highly interactive e-learning module improves NIHSS knowledge and skills more efficiently than the traditional didactic video amongst a mixed population of stroke unit, neurology and neurosurgery ward nurses, and physicians with different levels of experience and expertise.

Results of previous studies have shown that knowledge acquisition was higher in medical students than in paramedics [19]. This is probably due to a better understanding of the neurological system and of clinical testing, even though participants within both groups had no prior NIHSS knowledge. The current study is the first to test the impact of the e-learning module on a population with pre-existing NIHSS knowledge.

Further, we should also be able to determine the impact of these learning modalities on knowledge retention at one month. The decline in performance has been shown to be nonlinear; among participants displaying a significant decline in knowledge retention at 3 months, half of them present a significant decline already after 4 weeks [35]. This time interval has been recently used [36]. Electronic health (e-health) literacy was already high in some countries prior to the COVID-19 pandemic [37] and should have considerably increased in the wake of this crisis. Therefore, determining the most efficient asynchronous distance learning modality to help health care workers improve their NIHSS knowledge is both timely and relevant.

Given the importance the NIHSS score plays in the diagnosis, treatment decisions, and follow-up of stroke patients [1], it is essential to ensure that the NIHSS application skills are maintained at a high level. The study design should also help us gain knowledge of the current performance of neurology and neurosurgery nurses and physicians, prior to any teaching intervention, by virtue of the first (baseline) quiz. This could help determine the necessity of recurrent training in these highly specialized wards to maintain high performance in NIHSS application. There is little reason to believe that the potential decline in NIHSS application knowledge this study could reveal would be different in other settings or countries, and the results obtained should therefore be generalizable.

### 3.2. Strengths and Limitations

Both pathways of the study require approximately two to four hours to be completed, and participants will be asked to repeat the 50-question quiz after one month. There is currently a high level of fatigue amongst hospital staff related to the COVID-19 pandemic [38]. Even though the personnel and the different hierarchies express a high level of enthusiasm for this type of study, uncertainty remains regarding the number of participants who will engage in this study, and attrition might be high [39]. Furthermore, even though each participant will have their own login and password, we will not have any way to ascertain that the quizzes will be completed by one person and not a group of colleagues. However, we will emphasize the single-person performance aspect of this study in the instruction page, and believe that the risk of such bias is low. Although we will specify that no external resources are allowed, we will have no way to verify that these have not been used (e.g., detailed NIHSS form or internet page with explanations). The temptation to do so can be important, especially when study subjects desire to prove that their baseline NIHSS knowledge is high. This could bias the results with an over-assessment of baseline knowledge. The heterogeneity of English and French proficiency among nurses and physicians of both hospitals could have an impact on the results of the study. The e-learning is entirely in French, and Dr. P. Lyden’s [20] original video is in English. To minimize this effect, the entire video (be it the extracts in the e-learning or the full video) has been subtitled in French. Further, participants will also be asked about their language proficiency in the initial demographic questionnaire, and the distribution assessed among groups. Finally, using a 50-question quiz to determine NIHSS knowledge cannot be considered as entirely representative of the actual clinical application of this scale. The video vignettes have, however, been validated in prior studies and should therefore represent an acceptable surrogate outcome [18,19]. Moreover, reusing the same 50-question quiz could lead to an improvement from one time to another due to a “priming effect” of the first questionnaire. However, this effect should be smoothed out given the randomization as it should occur similarly in both groups.

## Figures and Tables

**Table 1:** First questionnaire.

Page	Field	Original Question	English Translation
1	Demographics	Age	Age ^a^
		Genre	Gender ^b^
		Années d’expérience clinique au total	Number of years of total clinical experience ^a^
		Années d’expérience clinique dans un service de neurologie et/ou de neurochirurgie	Number of years of clinical experience in a neurology and/or neurosurgery ward ^a^
		Maîtrise du Français	French proficiency ^c^
		Maîtrise de l’Anglais	English proficiency ^c^
		Dans quel service travaillez-vous principalement: Unité Cérébrovasculaire—surveillance continue Unité Cérébrovasculaire-étage Etage de neurologie Soins intermédiaires de neurologie Etage de neurochirurgie Soins intermédiaires de neurochirurgie Autre	In which ward do you most frequently work: ^b^ Stroke unit-surveillance unit Stroke unit-regular ward Neurology ward Neurology intermediate care unit Neurosurgical ward Neurosurgical intermediate care unit Other
2	Prior NIHSS knowledge	Avez-vous effectué une formation interne du service pour apprendre à faire une évaluation NIHSS?	Have you completed an in house NIHSS training? ^b^
		Avez-vous complété une formation certifiante officielle NIHSS?	Have you completed the official NIHSS certification course? ^b^
		Nombre d’années de pratique avec l’échelle NIHSS	Number of years of clinical experience with the NIHSS scale ^a^
		Fréquence d’application du NIHSS: Plusieurs fois par jour Environ 1 fois par jour Environ 1 fois par semaine Environ 1 fois par mois Très rarement ou jamais	NIHSS use frequency: ^b^ Many times per day Circa once a day Circa once a week Circa once per month Almost never or never
		Je me sens à l’aise par rapport à l’application du NIHSS	I feel comfortable using the NIHSS ^c^

^a^ Regex: regular expression validation. ^b^ MCQ: multiple-choice question (only one answer accepted). ^c^ 5-point Likert scale.

**Table 2:** Post-course questionnaire.

Page	Field	Original Question	English Translation
1	Prior NIHSS learning method exposition	Aviez-vous déjà suivi cette méthode de formation au NIHSS?	Had you already been exposed to this NIHSS learning method in the past? ^a^
		Quand avez-vous suivi cette formation? Il y a moins d’un mois Il y a moins de 6 mois Il y a moins d’un an Il y a plus d’un an Je ne me souviens pas	When did you undergo this training? Less than a month ago Less than 6 months ago Less than one year ago More than one year ago I do not remember
	Current training-context	Dans quel cadre avez-vous suivi cette formation? Demandée par mon service Trouvée cette formation sur internet par hazard Information recue par email	In which context did you undergo this training? Required by my employers Found it online by chance Information received via email
	Current training-satisfaction	Comment évaluez-vous le niveau de difficulté global de cette formation?	How would you access the overall difficulty level of this training? ^b^
		Que pensez-vous de la durée de cette méthode de formation?	What do you think about the duration of this training? ^b^
		Quel est votre niveau de satisfaction concernant la méthode de formation?	What is your level of satisfaction regarding the learning method? ^b^
		Recommanderiez-vous cette méthode de formation à vos collègues?	Would you recommend this training method to your colleagues? ^b^
		Avez-vous des commentaires supplémentaires?	Do you have any additional comments?

^a^ MCQ: multiple choice question (only one answer accepted). ^b^ 5-point Likert scale.

## Data Availability

Not applicable.
